# Canonical and alternate mechanisms that regulate ubiquitylation by the E3 ligase parkin

**DOI:** 10.1042/BST20253050

**Published:** 2025-08-18

**Authors:** Nicoletta T. Basilone, Viveka M. Pimenta, Gary S. Shaw

**Affiliations:** Department of Biochemistry, The University of Western Ontario, London, ON, N6A 5C1, Canada

**Keywords:** mitochondrial dysfunction, parkin, Parkinson’s disease, protein structure, ubiquitin ligases

## Abstract

Parkin, a Ring-InBetweenRING-Rcat E3 ubiquitin ligase, plays a vital role in the clearance of damaged mitochondria (mitophagy) by ubiquitylating a broad spectrum of mitochondrial proteins. Mutations in the PRKN gene alter parkin ubiquitylation activity and are a leading cause of early-onset Parkinsonism, underlining its critical function in maintaining mitochondrial homeostasis. The structures, substrates, and ubiquitylation mechanisms used by parkin in mitophagy are well established. Yet, early studies as well as more recent proteomics studies identify alternative substrates that reside in the cytosol or other cellular compartments, suggesting potential roles for parkin beyond mitophagy. In addition to its well-documented activation via S65 phosphorylation, numerous other post-translational modifications (PTMs) have been identified in parkin. Some of these modifications have the potential to serve key regulatory mechanisms, perhaps fine-tuning parkin activity or potentially signaling the involvement in alternative cellular pathways beyond mitochondrial quality control. This review examines the canonical mechanism of parkin-mediated ubiquitylation while also exploring alternative regulatory influences that may modulate its enzyme activity. By analyzing emerging evidence on PTMs including phosphorylation, acetylation, ubiquitylation, oxidation, and interaction with alternative activating molecules, we highlight the broader functional landscape of parkin and its implications for cellular stress response.

## Introduction

Mutations in the gene *PRKN*, many causative for different forms of early-onset Parkinson’s disease (PD), were discovered more than 25 years ago [1]. Early studies showed that genetic substitutions in the protein product—parkin, caused protein instability or aggregation, altering its function in the ubiquitin (Ub)-mediated proteolysis pathway [2–7]. Clinically, these defects in parkin manifest in the loss of dopaminergic neurons in the substantia nigra region of the brain, a hallmark of PD [8]. Since these initial reports, multiple studies have elucidated structures, mechanisms of ubiquitylation, and substrates, providing important insights into the biological function of parkin and its role in PD progression.

**Figure 1:**
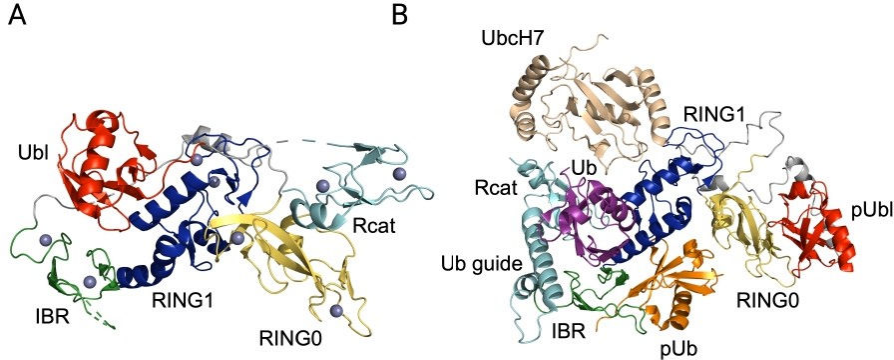
Autoinhibited and transthiolation states of parkin. (**A**) The autoinhibited structure of parkin (PDB: 5C1Z) [25] showing the positions for the Ubl (red), RING0 (yellow), RING1 (blue), IBR (green), and Rcat (cyan) domains. The repressor element (REP) is shown as the small gray helix above the RING1 domain. In the structure, the ubiquitin-like (Ubl) domain is positioned against the RING1 domain blocking the site for E2~Ub recruitment and the RING0 domain interacts with the Rcat domain occluding of the catalytic cysteine (C431). The linker region connecting the Ubl and RING0 domains and a portion of the tether between the IBR and Rcat domains are absent from the structure. (**B**) AlphaFold model (ma-1fhux, modelarchive.org) of the transthiolation complex for phosphorylated parkin bound to pUb (orange) and UbcH7–Ub (wheat, purple) [26]. A similar AlphaFold model has subsequently been reported [27]. The Rcat domain is recruited to the conjugated Ub in the UbcH7–Ub complex by formation of a Ub-guide helix. This provides a platform for Ub transfer to the Rcat domain forming a Rcat–Ub conjugate as shown in NMR (PDB: 9C5E) experiments [26].

Ubiquitylation involves the passage of the small protein Ub through E1, E2, and E3 enzymes, ultimately linking the C-terminus of Ub to the ε-NH_2_ group of lysine residues on a substrate [9]. The process is complex due to large numbers of E2 and E3 enzymes that give rise to thousands of possible E2/E3 permutations. In addition, different types of E3 ligases exist that dictate how Ub is passed on to a substrate. RING E3 ligases are Zn^2+^-binding scaffolds that co-ordinate Ub transfer directly from an E2 enzyme to a substrate [10]. Alternatively, HECT E3 enzymes recruit E2 enzymes and use a catalytic cysteine residue to form an E3~Ub thioester prior to transferring their cargo to a substrate [11]. Parkin is a mainly cytosolic, ubiquitously expressed E3 Ub ligase that has a higher abundance in the brain. Initial sequence analyses proposed that parkin is a RING E3 ligase enzyme due to the regular pattern of Zn^2+^-binding cysteine residues, typical of RING enzymes [12,13]. Experiments established that parkin functions with multiple E2 enzymes including UbcH7, UbcH8, and Ubc13 to ubiquitylate itself (auto-ubiquitylate) and many different cytosolic and nuclear proteins including SEPT5 (CDCrel-1), synphilin-1, GPR37 (PAEL-R), and ZNF746 (PARIS) [14–17]. Genetic substitutions in parkin showed multiple effects including aggregation, changes in stability, and misfolding that generally decrease auto-ubiquitylation [2,5,18]. Despite these successes, initial experiments were limited by an incomplete knowledge of the structure of parkin and how this might influence its function. Subsequently, early structural studies showed that parkin is more complex compared with traditional RING E3 ligases [19–24]. While parkin contains a canonical RING domain (RING1), it also possesses a C-terminal Rcat domain that contains a catalytic cysteine residue, akin to HECT E3 ligases. In addition, N-terminal ubiquitin-like (Ubl), RING0, and InBetweenRING (IBR) domains (Figure 1), not found in RING or HECT E3 enzymes, make up the remainder of the enzyme. Pioneering biochemical experiments showed that parkin lacking the Ubl domain is more active than the full-length enzyme in ubiquitylation reactions. This provided early evidence, prior to the availability of three-dimensional structures, that parkin exists in an auto-inhibited conformation [28], which requires a stimulus to modulate its ubiquitylation activity. Along with structures of parkin and related enzymes, these observations paved the way for experiments that showed parkin ubiquitylation combines elements of both RING and HECT E3 ligase mechanisms [29]. These features place parkin, along with enzymes such as ARIH1 [30], HOIP [31], and RNF216 [32] in the unique class of RING-InBetweenRING-Rcat (RBR) E3 ligases [33].

It is now widely recognized that parkin has important roles in mitophagy through its recruitment to mitochondria and ubiquitylation of a broad spectrum of mitochondrial proteins as a signal for removal of these damaged organelles [34,35]. Multiple proteomics experiments conducted under simulated cellular oxidative stress conditions have shown hundreds of possible substrates are ubiquitylated [36,37]. Yet many target proteins of parkin ubiquitylation were initially identified in the absence of oxidative stress. More recent proteomics experiments have identified substrates that are cytosolic or located in other compartments not clearly associated with mitophagy [38]. In this review, we describe the canonical mechanism for ubiquitylation by parkin and discuss other post-translational modifications (PTMs) or co-factors that could contribute to additional regulatory mechanisms for ubiquitylation by this unique E3 ligase.

## Canonical mechanism of parkin leading to mitophagy

The recruitment of parkin to the outer mitochondrial membrane (OMM) is an initial step for mitophagy, the canonical pathway for parkin function. Under basal conditions, parkin is cytosolic and exists in an auto-inhibited conformation with minimal ubiquitylation activity (Figure 1). This arises due to interactions between the Ubl and RING1 domains that hinder E2-conjugating enzyme recruitment and the inaccessibility of the catalytic cysteine residue (C431) in the Rcat domain that is unable to accept Ub from an E2~Ub conjugate [22,25,39]. Disruption of the mitochondrial membrane potential using decoupling chemicals such as carbonyl cyanide m-chlorophenylhydrazone (CCCP) and oligomycin/antimycin A causes the recruitment of parkin to mitochondria [40]. An accumulation of reactive oxygen species (ROS) at the OMM stabilizes and activates the serine/threonine kinase PINK1 [41–43]. This leads to the phosphorylation of Ub and polyubiquitin chains that have been pre-assembled on OMM-linked proteins such as Miro1/2, mitofusin1/2, and CISD1. The specific phosphorylation at S65 in ubiquitin (pUb) by PINK1 [44–46] promotes the translocation of parkin from the cytosol to mitochondria [36,40–42,47], facilitated by the tight binding of pUb to the RING0/RING1 interface of parkin [25,39,48]. Binding of pUb causes a change in parkin’s structure, moving the IBR domain away from the Ubl domain [25,39,48] that allows E2 binding to the RING1 domain and opens a cavity for Ub binding from an E2~Ub conjugate [49,50]. The shift in IBR location also allows further phosphorylation by PINK1 at S65 of parkin’s Ubl domain [51,52] (Figure 1). Structures lacking the Rcat domain show these dual phosphorylation events result in conformational changes that relocate the phosphorylated Ubl (pUbl) domain to a basic patch (K161, R163, K211) on the RING0 domain surface [53,54]. It is proposed that re-binding of the pUbl domain disengages the Rcat domain, making its catalytic cysteine available to accept Ub from an E2~Ub conjugate, forming a parkin intermediate. A recent NMR structure of the Rcat–Ub complex, that exemplifies this intermediate, shows the conjugated Ub binds to the tether region, just prior to the ordered Rcat structure, unresolved in all prior structures, and forms a transient α-helix (a ‘guide helix’) [26]. The Rcat–Ub structure is supported by chemical crosslinking/mass spectrometry experiments that place the donor UbcH7 enzyme close to the acceptor Rcat domain in the transthiolation complex [26] (Figure 1). Similar arrangements have been identified for other RBR E3 ligases such as HOIP and ARIH1 [31,55]. These observations indicate that E2~Ub binding to parkin may be a driving force for Rcat relocation and Ub transfer by providing a hydrophobic surface in Ub for the masked guide helix in the parkin tether region. These complicated structural events that occur in response to mitochondrial oxidative damage give rise to an estimated 4000-fold increase in ubiquitylation activity by parkin compared with its auto-inhibited state [56]. Consistent with this, parkin knock-out cells are defective in mitophagy [41,57]. Details of this pathway at the cellular and structural levels have been described in multiple excellent reviews [34,35,58–60].

## Alternate modes that regulate parkin activity—phosphorylation

**Figure 2:**
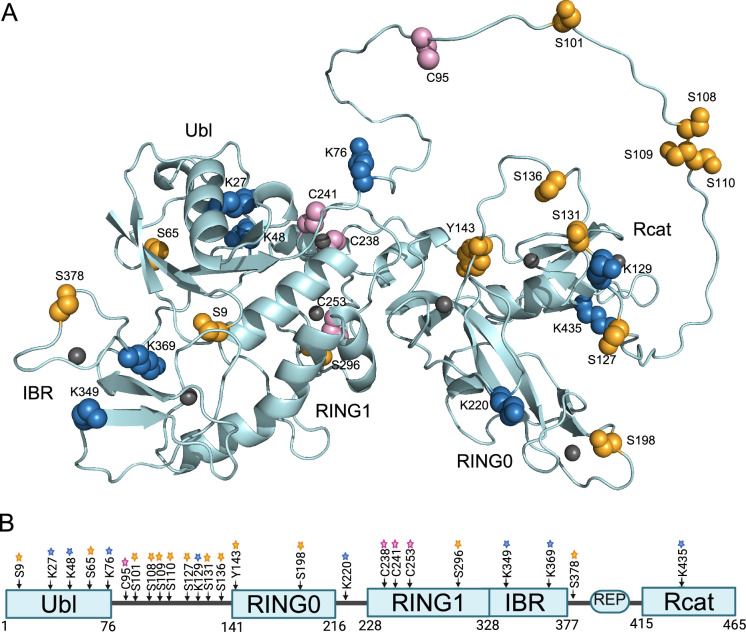
Post-translational modifications of parkin. (**A**) Structure of auto-inhibited parkin (PDB: 5C1Z) [25] showing sites of phosphorylation (orange), oxidation (pink), and lysine modification (blue) as spheres. The linker and portions of the tether region are either not observed in crystal structures or absent from protein constructs but are modeled here using Chimera [61] to illustrate positions of modifications. Lysine modifications include acetylation (**K76, K129, K220, K349**), ubiquitylation (**K27, K48, K76, K129, K220, K349, K435**), NEDDylation (**K76**), and ISG15ylation (K349, K369). Zn^2+^ ions are shown as gray spheres. (**B**) Domain illustration for parkin showing post-translational modifications depicted in (**A**). Created in BioRender. Basilone, N. (2025) https://BioRender.com/sepbsyy.

In addition to PINK1-mediated S65 phosphorylation of parkin, which occurs exclusively at the OMM, 12 supplemental phosphorylation sites are reproducibly observed in the literature (Figure 2). Additional sites determined from mass spectrometry-based proteomics experiments are available (phosphosite.org) but remain to be validated. A broad series of protein kinases responsible for parkin phosphorylation have been identified from *in vivo* and *in vitro* experiments including AMPK (S9) [62], CaMK2 (S9, S198) [63], ULK1 (S108, S109, S110) [64], mTORC1 (S127) [65], p38 MAPK/Dyrk1A (S131) [66–68], Cdk5 (S131) [69], c-Abl (Y143) [70], and Plk1 (S378) [71]. At least five sites (S101, S131, S136, S296, and S378) in parkin are constitutively phosphorylated in HEK293 and/or neuronal SH-SY5Y cell types [72,73]. Increased levels of phosphorylated S101 are detected in the caudate region of some PD patients [72].

Seven phosphorylation sites lie within the 65-residue unstructured linker that joins the Ubl and RING0 domains (Figure 2). A recent study identified S108, S109, and S110 as substrates of ULK1, an upstream kinase to the mitophagy pathway that, like PINK1, is activated upon mitochondrial stress [64]. Phosphorylation at S108 occurs rapidly in the cytosol, preceding PINK1 phosphorylation and recruitment of parkin to the OMM [64]. The phosphorylation at S108, S109, and S110 may be important for translocation to the OMM, and subsequent phosphorylation at S65 of parkin by PINK1, as substitution of all three residues, delays this association. These three phosphorylation sites along with S101 are found within or neighboring the activating element (ACT) in parkin (L102-L107), a conserved sequence in the linker region that stabilizes a hydrophobic surface on the RING0 domain observed in a structure of Rcat-deficient parkin [53]. The structure shows that S101 and S108 are mostly solvent accessible, so it is not clear how phosphorylation of these would affect parkin’s ubiquitylation activity. The upstream kinases to ULK1, AMPK targets S9 [62] while CaMK2 targets both S9 and S198 of parkin [63], in a PINK1-independent manner, promoting recruitment to synaptic vesicles and modulating the ubiquitylation of synaptojanin-1. Structurally, phosphorylation at S9 is suggested to alter the Ubl interface with the IBR domain in auto-inhibited parkin. Phosphorylation of S198 might be expected to interfere with Zn^2+^ binding, due to its proximity to cysteines in one of the metal-binding sites in the RING0 domain. Downstream of the ACT element, the conserved GLAVIL motif (G118-L123) has been shown to be important in substrate recruitment by parkin [74,75]. Phosphorylation of neighboring residues (S127, S131) decreases the basal ubiquitylation activity of parkin [65,69], although it is not known what the effect is on substrates. Although S131 phosphorylation does not appear to alter subsequent PINK1 phosphorylation of S65 in parkin, it is unclear whether S131 phosphorylation affects the ubiquitylation activity of the fully activated E3 ligase.

The remaining two phosphorylation sites lie in the RING1 (S296) and immediately following the IBR (S378) domains. As described above, these sites are constitutively phosphorylated but rapidly turned over due to dephosphorylation in vivo. Polo-like kinase (Plk1)-driven phosphorylation of S378 during mitosis shows enhanced ubiquitylation by parkin of mitotic substrates such as cyclin B1 and securin in a PINK1-independent fashion [71]. Phosphorylation of S296 and S378 (also S101, S131, S136) is reduced in response to cellular protein folding stresses [73]. While the affects of phosphorylation on parkin’s ubiquitylation activity are uncertain, complementary overexpression of the kinases responsible for these sites in HEK293T cells results in significant protein aggregation involving parkin. This would indirectly compromise overall activity in cells [72].

Phosphorylation of Ub also affects parkin activation. While pUb^S65^ is well known to activate parkin in the canonical mitophagy pathway, Ub phosphorylated at other sites including T12 and S57 can also affect the ubiquitylation efficiency of the E3 ligase [76]. Multiple kinases, including MARK1-4 and PKA have been shown to phosphorylate S57 in Ub [77,78]. *In vitro*, pUb^ S57^ is hyperactive for parkin auto-ubiquitylation in comparison with pUb^S65^ [76]. While this might provide a potential means of PINK1-independent activation, it remains to be clarified how ubiquitylation is altered with substrates and in a cellular context. Multiple *in vitro* studies show that auto-ubiquitylation by parkin is inhibited when phosphorylated Ub (S12, S20, S65) is used as the sole Ub source [36,79,80].

## Alternate modes that regulate parkin activity—acetylation and ubiquitylation

Acetylation and ubiquitylation of enzymes in ubiquitylation pathways can have dramatic effects on activity and outcomes [81,82]. Parkin has 18 lysine residues that are potential targets for acetylation and ubiquitylation that might alter its regulation. Mass spectrometry has identified seven lysine residues (K27, K48, K76, K129, K220, K349, and K435) as sites for ubiquitylation under different conditions [21,83,84] (Figure 2), although the outcomes of these PTMs are unknown. Parkin is known to auto-ubiquitylate itself at sites K27, K48, and K76 upon CCCP treatment, likely through K6- and K11-linked chains [36,37,83]. These chain types are also assembled on other substrates. The deubiquitylating enzyme (DUB) USP8 regulates parkin by removing K6-linked Ub chains from the E3 ligase [83]. Knockdown of USP8 results in reduced parkin recruitment to mitochondria, whereas overexpression of Ub^K6R^ rescues recruitment, indicating that removal of K6-linked Ub chains from parkin is a requirement for its efficient translocation to mitochondria. This may be related to the fact that K6-linked chains are known to protect substrates from proteasomal degradation or direct proteins to alternative paths. Other DUBs have been shown to work directly on parkin including USP33, which shows K435 as its primary site of activity [85]. Alternatively, USP33 affects K48 and K63-linked chains, and its knockdown results in improved parkin translocation to depolarized mitochondria, protecting SH-SY5Y cells from neurotoxin MPTP-induced apoptotic cell death [85]. On the other hand, N-terminal solubility/affinity tags are known to alter auto-ubiquitylation by parkin [86]. For example, parkin carrying an N-terminal maltose-binding protein (MBP) preferentially adds monoubiquitin to the MBP moiety rather than parkin itself [87].

One ligase found to ubiquitylate parkin is MITOL, which assembles K48-linked chains at K220 of the RING0 domain and targets S65 phosphorylated parkin for degradation [84]. In most cases, however, it is not clear how ubiquitylation alters the function of parkin. It was recently shown that ubiquitylation at K27 and subsequent phosphorylation of this Ub leads to increases in auto-ubiquitylation of parkin [88]. It is proposed this may be a result of association of parkin molecules at the OMM via the K27-linked pUb moiety [88]. This observation appears distinct from the K27N disease variant of parkin. While this variant cannot be ubiquitylated at K27, the asparagine substitution causes instability in the Ubl structure and shows conflicting impacts on ubiquitylation by the E3 ligase [28,88].

Acetylation is known to modulate autophagy activity, as acetylation of Ub results in the absence and reduced elongation of chains [89]. In addition, lysine acetyltransferase-8 (KAT8) modulates PINK1-parkin-dependent mitophagy by attenuating *PINK1* gene transcript levels, protein levels, and subsequent PINK1 phosphorylation of Ub and parkin [90]. Likewise, PD loss-of-parkin genetic mutations promote acetylation of microtubules in DA neuron cell bodies and fibers, resulting in decreased stability and transport [91]. It was recently reported that parkin can be acetylated at residues K76, K129, K220, K349, and K408 using suberoylanilide hydroxamic acid (SAHA) as a deacetylase inhibitor [92] in HEK293 cells. This study used lysine to arginine substitutions to show K129, K220, and K349 are the main sites of acetylation by acetyl-CoA acetyltransferase (ACAT1). In the presence of the SAHA inhibitor, recruitment of parkin to mitochondria is improved. While an increase in S65 phosphorylation in parkin is noted, it is not clear whether similar phosphorylation changes occur for Ub [92]. Enhanced ubiquitylation of mitochondrial proteins and removal of damaged mitochondria occurs, indicating that acetylation of parkin may provide an additional level of regulation for mitophagy.

## Alternate modes that regulate parkin activity—oxidation

Parkin recruited to mitochondria and activated under oxidative stress is highly sensitive to oxidative modification [4,93,94]. Compared with other human proteins, parkin features an unusually high proportion of cysteine residues found in all regions of the protein (7.5%), including 28 that co-ordinate eight Zn^2+^ ions within its C-terminal RING0, RING1, IBR, and Rcat domains [95,96]. Cysteine residues are the most oxidation-prone sites in proteins, where thiols can be irreversibly oxidized to sulfinic (SO_2_H) and sulfonic acids (SO_3_H) or form reversible disulfide bridges [97]. While these types of modifications are rare in the cytosol, many cytosolic proteins have been identified with oxidative modifications under basal conditions [98–101]. Such modifications alter the chemical properties of cysteine and can disrupt the overall protein structure and function.

For parkin, the close relationship between cysteine modifications and ubiquitylation activity is highlighted by the sensitivity of PD substitutions to Zn^2+^-binding cysteine residues. Substitutions to Zn^2+^-coordinating residues C150G, C166A, and C212Y (RING0); C238W, C253Y, and C289G (RING1); and C418R and C441R (Rcat) in parkin often result in aggregation or instability with altered ubiquitylation efficiency [6,7,87,102–105]. Surprisingly, some cysteine substitutions (C323S and C457S) have limited impact on parkin translocation to the OMM while others that modify the catalytic site (C431F and C431S) have significant defects [36] that frequently can be traced to insolubility (C212Y, C289G, C418R, and C441R) [41]. A comprehensive mutational analysis, in which all parkin cysteines were individually substituted to alanine and extracted from SH-SY5Y neuroblastoma cells, demonstrated that solubility is least affected by the replacement of non-Zn^2+^-binding cysteines (C59, C95, C268, C323, C451), including the catalytic cysteine (C431) [102]. Truncation of the C-terminal region of parkin (N464–V465) promotes aggregation throughout the cytosol in SH-SY5Y cells, presumably through the loss of Zn^2+^-co-ordinating C457 and H461 [4]. The ligase activity of parkin is tightly regulated by each of its domains, so it would stand to reason that oxidation or substitution of cysteine residues that alter Zn^2+^ binding will disrupt proper folding and lead to insolubility and decreased ubiquitylation activity.

Isolated brain tissue lysates of PD patients show increased parkin oxidation and insolubility compared with age-matched controls [94]. Age-dependent accumulation of oxidative stress results in parkin oxidation, as parkin isolated from neurologically healthy, aged human cortices includes oxidation at C95 and C253 [106]. Several other modifications result from the oxidation of dopamine that forms adducts at multiple sites including C95 and C238 in aged brain tissue (Figure 2). In SH-SY5Y cells, oxidation shows initial increases in auto-ubiquitylation by parkin followed by a return to limited or decreased activity [94]. Oxidation-induced auto-ubiquitylation is accompanied by aggregation and losses of substrate ubiquitylation, while the addition of reducing agents partially restores parkin E3 function [107,108]. These findings indicate that ubiquitylation by parkin is not directly perturbed by early oxidation events but becomes more vulnerable as oxidation progresses. Oxidized parkin becomes sequestered in aggregates throughout the cytosol, nucleus, and mitochondria, suggesting that ubiquitylation at the OMM is hindered [94]. The association between oxidation and parkin aggregation is intriguing, since Lewy bodies (LBs) containing insoluble, aggregated protein are a well-known hallmark of sporadic PD and oxidative stress is thought to contribute to their formation. Studies of human brain tissue have shown a decline in parkin solubility that positively correlates with both age and a rise in oxidative stress [106]. Further, it has been shown that parkin, Ub, and UbcH7 localize to the core of LBs in PD brains, suggesting that parkin ubiquitylation and oxidation are not mutually exclusive events in PD pathology [109]. It has been proposed that parkin may have a protective redox role, neutralizing ROS by becoming oxidized itself, as multiple studies show that parkin can reduce ROS concentration in neurons [107,110].

Of the 35 cysteine residues in human parkin, nearly all are capable of being oxidized *in vivo*/*in vitro* under increasingly harsh oxidizing conditions (i.e., high concentrations of oxidant, longer exposure times). Tightly controlled age- and time-dependent experiments are necessary since initial oxidation that results in unfolding of the protein will inevitably accelerate the oxidation at other sites. Nevertheless, two intensive studies have identified cysteine residues in purified parkin susceptible to oxidation using the natural oxidant hydrogen peroxide (H_2_O_2_) [94,106]. These experiments focused on irreversible (–SO_2_H or –SO_3_H) and reversible (–S–S–) modifications determined from mass spectrometry experiments. Although some differences are noted in these reports, both reproducibly identified C95 (linker) and C238, C241, and C253 (RING1) are oxidized under pathological doses (10–20 μM) of H_2_O_2_ or in brain tissue (Figure 2). Should these oxidation events result in a soluble E3 ligase, alteration of cysteine residues in the RING1 domain would undoubtedly compromise binding by the E2~Ub conjugate and negatively affect ubiquitylation by parkin.

## Can other Ubl proteins alter parkin activity?

The interaction of parkin with pUb is a main driving force to relieve auto-inhibition. Recently, it was observed that mitochondrial substrates such as Miro1 and CISD1, tethered to phosphorylated Ub, are efficiently ubiquitylated even in the absence of parkin phosphorylation by PINK1 [111]. This may represent proximity-based, or substrate-assisted, ubiquitylation of a substrate once a pUb is bound to the E3 ligase. Alternatively, this may result from binding of a secondary pUb, formed from PINK1 phosphorylation of a ubiquitylated substrate at the OMM, to the RING0-binding site (K161, R163, K211), shown to interact with pUbl in crystal structures. In trans, this region has a higher affinity for pUb (400 nM) than for pUbl (1 μM) although it might be expected that the local concentration effect would favor pUbl binding in cis [112]. A recent crystal structure shows that binding of two pUb molecules at the traditional RING0/RING1 cleft (K151, H302, R305) and the relocated pUbl site on the RING0 domain is consistent with previous ubiquitylation assays that show increased substrate ubiquitylation in the absence of parkin phosphorylation [113]. This work has paved the way for ‘molecular glues’ that make use of pUb binding to the RING0 pocket. For example, tetrahydropyrazolo-pyrazine compounds developed by Shlevkov et al. [114] mimic the ACT element in parkin, making contact with a pUb (as opposed to pUbl) and the RING0 domain to enhance pUb affinity and parkin activation [115]. These molecules work exclusively with the interaction of two pUb molecules, bypassing the need for PINK1 phosphorylation of parkin and providing a potential rescue pathway for genetic PD-based substitutions in the Ubl domain that limit its folding, phosphorylation, and binding.

The observation of interacting proteins and possible ubiquitylated substrates for parkin that are not associated with the mitochondria suggests that other activating molecules may exist in the cytosol or other organelles. As described earlier, alternate phosphorylated forms of Ub are capable of activating parkin [76]. In addition, there are a large group of Ubl proteins that may be capable of allosterically activating parkin like pUb or pUbl. For example, NEDD8 has 58% sequence identity and 80% structural similarity to Ub including a hydrophobic ‘I44 patch’ and a S65 residue that PINK1 can phosphorylate *in vitro*, albeit much less efficiently than for Ub [45,116]. A recent crystal structure shows pNEDD8 binds to parkin at the RING0/RING1 cleft in a similar fashion as pUb and with improved affinity [117]. Consistent with this finding, pNEDD8 also increases parkin auto-ubiquitylation and Miro1 ubiquitylation activity *in vitro* [116,117]. While there is some overlap in localization between parkin and NEDD8 in the nucleus [118–120], a kinase specific for NEDD8 phosphorylation remains to be identified. Similarly, the Ubl protein ISG15 has comparable serine residues (S69 and S146) to S65 in Ub, and SUMO1 has a structurally similar acidic patch in this region (E83-D86) that could activate parkin. *In vitro*, SUMO-tagged parkin lacking its Ubl and linker regions leads to increased auto-ubiquitylation activity [20], although it is well documented that N-terminally tagged parkin can produce ‘false-positive’ activation [86]. Further, there are no reports of phosphorylation for ISG15 and noncovalent interactions between parkin and ISG15 or pISG15 have yet to be identified.

Not surprisingly, several of these Ubls are already known to modify E3s and modulate their activity, function, or localization through covalent modification [66,121–124]. NEDDylation of parkin at K76 increases parkin substrate ubiquitylation while not affecting subcellular localization or solubility [122,123]. It is proposed that the enhanced activity is facilitated by improved E2 and substrate (p38, TRAF2) recognition [123]. Parkin can be modified with ISG15 by the E3 ligase HERC5, primarily at residues K349 and K369 in the IBR domain. These modifications increase parkin auto-ubiquitylation activity through suspected relief of autoinhibitory interactions between the Ubl domain and the C-terminus of parkin [66]. It is notable that ISG15ylated residue K349 has been observed to be ubiquitylated previously [21] and is located near residues Q347 and C349 used to covalently link pUb to parkin to form a partially activated E3 ligase [48,50]. At this position, it is possible that ISG15 modification may function much like pUb binding to activate parkin. Further structural experiments (NMR or crystallography) are needed to validate any domain rearrangement.

Parkin works with the E1-activating enzyme UBA6 and E2-conjugating enzyme USE1 to add the Ubl protein FAT10 (FAT10ylate) to itself at two or more sites and to the mitochondrial protein mitofusin-2 [121]. Under oxidative stress, FAT10ylation of parkin delays its translocation to mitochondria and impairs ubiquitylation. Auto-FAT10ylation occurs in the cytosol where parkin is normally auto-inhibited in the absence of oxidative stress or PINK1 involvement. While this suggests an alternate activation pathway exists to activate parkin and modulate FAT10ylation activity, it remains to be determined how this occurs. It is also not clear what mechanisms might stimulate activation of parkin, although cytokine factors appear to improve FAT10ylation. The sites of FAT10 modification on parkin remain to be identified and may offer clues to this activity.

One study shows SUMO1 interacts with parkin noncovalently to promote translocation to the nucleus and increase auto-ubiquitylation; however, the impact this interaction has on substrate ubiquitylation is not yet resolved [125].

## Summary and conclusions

The response of parkin to oxidative stress and its subsequent ubiquitylation of mitochondrial proteins that trigger mitophagy is firmly established. However, parkin also localizes to other cellular compartments including nuclei, Golgi network, and secretory vesicles [125–128] and exhibits ubiquitylation of substrates involved in gene regulation (PARIS, HIF-1α) [15], endoplasmic reticulum stress (PAEL-R) [16], synaptic vesicle recycling (synaptojanin-1) [63], and cell trafficking (CDCrel-1, tubulin) [14,129,130]. It is not clear what stimulates the movement of parkin to these other organelles or how this might contribute to activate the E3 ligase from its auto-inhibited resting state, independent of PINK1. Perhaps the answer to parkin’s versatility lies in recent studies that show alternate interacting molecules or PTMs including phosphorylation, acetylation, and oxidation, which could promote distinct mechanisms for parkin’s ubiquitylation activity. Additional validation experiments including *in vitro* and *in vivo* substrate ubiquitylation assays (as opposed to auto-ubiquitylation) using distinct modifications and substrates, along with structural experiments to uncover how these modifications alter the structure of parkin’s auto-inhibited state, would help confirm the impact and mechanisms of some of the newly discovered PTMs on parkin activity. Mass spectrometry-based proteomic studies using stimuli other than oxidative stress may uncover regulatory events that trigger PTMs of parkin and modification of its substrates. These future experiments may clarify how parkin ubiquitylation activity is fine-tuned and will provide a more complete understanding of its interactive network.

**Perspectives**

- Mutations in the gene *PRKN*, which encodes the cytosolic E3 ubiquitin (Ub)-ligase parkin, are causative for early-onset Parkinson’s disease (PD). During oxidative stress, parkin is recruited to mitochondria where it ubiquitylates a broad spectrum of mitochondrial proteins to signal the removal of these damaged organelles as part of the mitophagy pathway.
- Parkin is inactive in the cytosol but is activated through binding to phosphorylated Ub at the outer mitochondrial surface and further phosphorylated by the kinase PINK1. Structural experiments have revealed the mechanisms and conformational changes in parkin needed to trigger Ub transfer, though recognition of substrates is only now being established.
- More than 25 auxiliary post-translational modifications (phosphorylation, acetylation, oxidation) have been identified in parkin. Future studies are needed to explore many of these alternate modifications to determine how these might modulate or fine-tune parkin regulation under different cellular conditions. This may reveal new mechanisms for parkin’s role in PD.

## Abbreviations

PD: Parkinson’s disease
PTMs: post-translational modifications
RBR: Ring-InBetweenRING-Rcat
ROS: reactive oxygen species
Ub: ubiquitin
Ubl: ubiquitin-like
